# Mercury Levels in Raccoons (*Procyon Lotor*) from the Warta Mouth National Park, Northwestern Poland

**DOI:** 10.1007/s12011-014-9962-2

**Published:** 2014-04-16

**Authors:** Natalia Lanocha, Elzbieta Kalisinska, Danuta I. Kosik-Bogacka, Halina Budis, Joanna Podlasinska, Ewa Jedrzejewska

**Affiliations:** 1 Department of Biology and Medical Parasitology, Pomeranian Medical University, Powstancow Wielkopolskich 72, 70-111 Szczecin, Poland; 2 Department of Health Education, University of Szczecin, Piastow 40B, 71-065 Szczecin, Poland; 3 Department of Environmental Management and Protection, Western Pomeranian University of Technology, Słowackiego 17, 71-374 Szczecin, Poland; 4 Warta Mouth National Park, Chyrzyno 1, 69-113 Górzyca, Poland

**Keywords:** Environmental pollution, Mercury, Northwestern Poland, Raccoon

## Abstract

This is the first report on mercury (Hg) levels in the liver, kidney, skeletal muscle, and brain of raccoon in Europe. It studied Hg concentration in 24 raccoons from the Warta Mouth National Park, northwestern Poland by atomic absorption spectroscopy (AAS). The highest total Hg concentrations in the raccoon were found in the liver (maximum, 18.45 mg/kg dry weight), while the lowest in the brain (maximum, 0.49 mg/kg dw). In adult raccoons, Hg concentrations in the liver, kidney, and brain were higher than in immature individuals (*p* < 0.001), while similar in skeletal muscle in both age groups. Our results are consistent with studies by other authors conducted in North America in areas with similar environmental conditions.

## Introduction

Mercury (Hg), in addition to lead and cadmium, is considered to be one of the most toxic trace metals. In 2005, global anthropogenic emissions of Hg to the atmosphere was estimated at 1,930 t, mostly in Asia (over 66 %), with much less in Europe and North America (8 % each) [1]. The total atmospheric emission of this element in Poland was estimated at almost 16 t in 2007 [2]. Biomagnified along the aquatic trophic chains, mercury reaches its highest concentrations in predatory fish, piscivorous birds, and mammals [3–5]. The estimated biomagnification results in low water Hg concentrations being multiplied in piscivores by 1 to 10 million times [6].

Mercury, especially methylmercury (MeHg), affects the function and development of the central nervous system in wildlife, resulting in a broad range of adverse health effects such as reproductive and behavioral problems. High Hg levels can be found in the habitats and food sources of many wildlife species. Mercury poisoning symptoms, mainly motor impairment, have been described in the American mink *Neovison vison* (Schreber, 1777), the Canadian otter *Lontra canadensis* (Schreber, 1777), and the red fox *Vulpes vulpes* (Linnaeus, 1758) [7–9].

Ecotoxicological research on Hg pollution relies to a great extent on the use of bioindicators. Among the mammals in North America, bioindicators often include local common species as the following: *N. vison*, *L. canadensis*, and the raccoon *Procyon lotor* (Linnaeus, 1758), while in Europe the native Eurasian otter *Lutra lutra* (Linnaseus, 1758). An indirect evaluation of Hg environmental pollution usually involves the determination of Hg concentration in the soft tissues of the animals, mainly in the liver and kidney [10–13].

The raccoon *P. lotor*, similar to the raccoon dog *Nyctereutes procyonoides* (Gray, 1834) and the American mink *N. vison*, is one of the most successful alien carnivores in Europe [14], being 1 of the 33 alien mammal species that have established self-sustaining populations on the continent [15]. The raccoon was brought to the continent in the 1930s, with feral raccoons in the 1950s and 1960s initiating free-ranging populations in Germany, mainly in its central areas and in Brandenburg, a region near the border with Poland [16]. In the 1980s, the raccoon appeared in western Poland, and then a significant influx of its population to Poland was observed in the years 1995–2004, when raccoons were observed near the Odra River in the Szczecin area and in the mouth of the Warta River. By the turn of the century, raccoons had been reported in 15 sites in the central lower Odra Valley and in the lower section of the Warta [17–19].

In contrast to North America, there is no data on Hg concentrations in the European populations of *P. lotor*, which often inhabits the environment around various water bodies, including large lowland rivers significantly polluted with Hg and other heavy metals, e.g., from industrial areas, and as such exposed to contaminated food. In North America, the raccoon is considered to be a suitable bioindicator of environmental pollution [20–22]. But so far, this mammal has not been used in Europe as an indicator organism in environmental studies on Hg pollution. Accordingly, the aim of this study was to determine total Hg concentrations in liver, kidney, semimembranosus muscle, and brain of raccoons originating from the Warta Mouth National Park (WMNP) in northwestern Poland.

## Methods

**Fig. 1:**
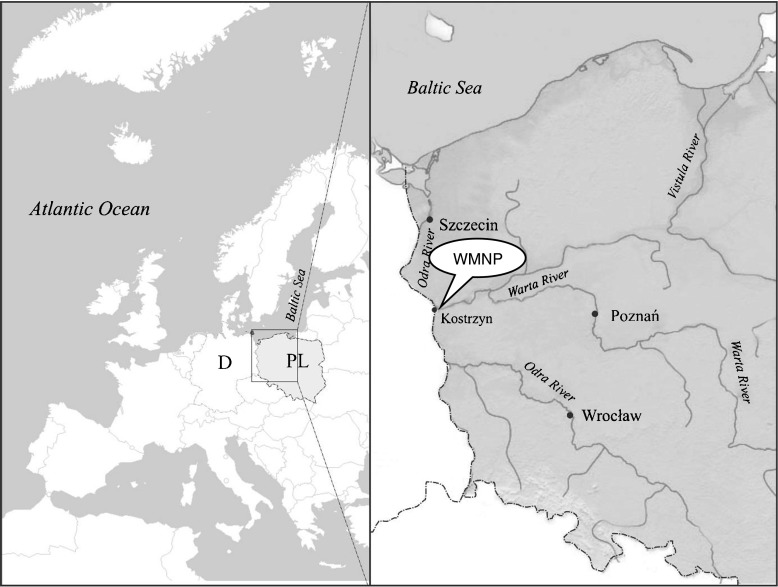
Location of study areas in northwestern Poland

The study area was the WMNP (80.7 km^2^), in the Lubuskie voivodship, western Poland (52°34′N, 14°43′E). These are floodplains of the lower section of the Warta River and its outlet to the Odra, a fragment of the Odra valley and an area between Kostrzyn and Słońsk, surrounded by flood embankments (Fig. 1). From autumn to spring, 50–100 % of the reservoir is usually flooded, with only small islands and hummocks remaining above the water [23]. The area forms a mosaic of flooded meadows, willow thickets (*Salix* spp.) and old river beds, shallow lakes, and land improvement canals with overgrown plants [24]. Forests cover only 0.82 km^2^ (1 % of the Park’s surface area), while arable land and water cover 26.62 km^2^ (~33 %) and 2.97 km^2^ (~4 %), respectively.

The Odra is the second largest river in Poland, constituting a large section of the Polish–German border. River sediments collected from the Odra and Warta Rivers in the vicinity of Kostrzyn contain elevated levels of heavy metals, including Hg [11, 25]. The concentration of Hg in the sediments of the Odra ranged from 0.25 to 1.49 mg/kg in Kostrzyn [25]. The main anthropogenic sources of heavy metals (including Hg) in the Odra River are the Upper Silesian coal mining and metallurgical district, and the Lower Silesian district (with capital in Wroclaw) nonferrous (especially copper) metallurgy (between Lubin and Glogow, from 310 to 393 river km). The Odra flows through the following Polish cities: Opole, Wroclaw (266 river km), Kostrzyn (617 river km), as well as some German cities, Eisenhuttenstadt (551 river km), Frankfurt (Oder), where major industrial enterprises are located, e.g., the steelworks of the Eisenhuttenkombinat-Ost [26].

This study was carried out on 24 raccoons (*P. lotor* Linnaeus, 1758), comprising 16 males and 8 females, found dead on the roads of the WMNP between 2009 and 2011. The raccoons were divided into two categories—immature individuals (*n* = 6) and adults (*n* = 17); the age of one individual could not be determined. Even though some organs were damaged, we collected 24 livers, 24 kidneys, 23 semimembranosus muscle (*musculus semitendinosus*), and 13 brains for chemical analysis. Carcasses were kept at −20 °C in a freezer. After dissection of the thawed carcasses, samples (about 10 g each) used for the determination of Hg were dried at 55 °C [10, 11]. Within 4–6 weeks, the samples were weighed (to 0.1 mg, Sartorius BP221S balance) three times to constant weight, which made it possible to determine weight-based sample water content. Subsequently, the samples were crushed in an agate mortar. Total Hg concentrations were determined using atomic absorption spectroscopy (AAS) at the Department of Environmental Management and Protection, West Pomeranian University of Technology, in Szczecin. The assays were run in an AMA 254 (Altach Ltd, Czech Republic) Hg analyzer in accordance with the procedure described by Kalisinska et al. [10, 11]; Hg detection limit for this device is about 0.01 ng Hg. The AMA 254 analyzer allowed determination of Hg in samples without having to perform prior mineralization in wet conditions. The analytical procedures were checked by determining Hg concentrations in samples of two reference materials—dogfish liver (DOLT-4 Dogfish Liver Certified Reference Material for Trace Metals, Canadian Irradiation Centre, Laval, Quebec) and bovine muscle (Reference Material 8414 Bovine Muscle Powder National Institute of Standards and Technology NIST, Canada). Our analysis of certified reference materials yielded total Hg concentrations of 2.59 ± 0.06 for DOLT-4 dogfish liver (*n* = 3) and 0.006 ± 0.0006 mg/kg dry weight (dw) 8414 NIST (*n* = 5). Corresponding values from the suppliers were 2.58 and 0.005 mg/kg dw, respectively. The recovery rates were 99.6 and 120 % for DOLT-4 and 8414 NIST, respectively.

Statistical studies were performed using Stat Soft Statistica 9.0 and Microsoft Excel 2007. We calculated the arithmetic means (AM), standard deviations of the AM (SD). The distribution of empirical data on Hg concentrations in the liver, kidney, muscle and brain diverged from the expected normal distribution, as shown by the Kolmogorov–Smirnov test with Lilliefors correction. Therefore, in comparisons of mean values of Hg concentration in the examined tissue, and, in order to compare the impact of gender and age, we used nonparametric Mann–Whitney *U* tests and Kruskal–Wallis tests (*p* < 0.05).

In addition, the Spearman’s rank correlation coefficients (*r*_*s*_) were determined for the relations between Hg levels in the materials collected from raccoons (liver, kidney, muscle, and brain) and age categories. Statistical significance was determined at *p* < 0.05 for all analyses.

## Results

Mercury concentrations were referred to both dw and wet weight (ww) of the tissue being analyzed. Mean percentages of water content in the liver, kidney, muscle and brain of the raccoon were 70.3 %, 78.4 %, 74.6 % and 78.9 %, respectively.

Data on Hg concentrations in the liver, kidney, skeletal muscle and brain of the raccoon are shown in Table 1. The highest mean Hg concentration was observed in the liver (2.99 mg/kg dw) and the lowest in the brain (0.14 mg/kg dw). Maximum liver Hg concentration exceeded 18 mg/kg dw in two samples. Similarly, in two cases, liver concentration was in the range of 6 to 10 mg/kg dw. In the studied materials, Hg mean concentrations can be arranged in the descending order liver > kidney > muscle > brain. High values of the coefficient of variation (up to 98.3 % for the muscle and 172 % for the liver) reflected significant differences in Hg concentrations in the examined tissues.

**Table 1:** Total mercury concentrations in liver, kidney, skeletal muscle, and brain of the raccoon age groups and gender (in mg/kg dw) and differences between Hg concentration in the examined raccoon tissues in this groups

Age or sex group, the number of individuals in each group (*n*) and parameters		Liver	Kidney	Muscle	Brain
im	*n*	6	6	5	5
	AM ± SD	0.06 ± 0.05	0.13 ± 0.17	0.32 ± 0.38	0.02 ± 0.01
	Med	0.04	0.06	0.11	0.01
	CV	80.4	133.6	117.0	72.2
	Min–max	0.03–0.15	0.03–0.48	0.01–0.79	0.009–0.04
ad	*n*	17	17	16	8
	AM ± SD	3.17 ± 4.66	2.47 ± 2.43	0.48 ± 0.45	0.21 ± 0.18
	Med	1.16	1.85	0.37	0.11
	CV	146.8	98.4	93.0	84.7
	Min–max	0.23–18.45	0.37–9.81	0.11–1.67	0.08–0.49
M	*n*	16	16	15	7
	AM ± SD	3.08 ± 4.88	2.24 ± 2.59	0.45 ± 0.46	0.14 ± 0.16
	Med	0.83	0.98	0.21	0.10
	CV	158.7	115.4	102.0	120.0
	Min–max	0.03–18.45	0.03–9.81	0.01–1.67	0.01–0.49
F	*n*	8	8	7	6
	AM ± SD	2.82 ± 5.98	1.71 ± 2.41	0.60 ± 0.58	0.14 ± 0.19
	Med	0.23	0.65	0.68	0.05
	CV	212.4	140.7	95.2	138.3
	Min–max	0.03–17.44	0.03–6.84	0.02–1.64	0.009–0.49
Total	*n*	24	24	22	13
	AM ± SD	2.99 ± 5.14	2.07 ± 2.50	0.50 ± 0.49	0.14 ± 0.17
	Med	0.78	0.90	0.29	0.08
	CV	172.0	120.6	98.3	123.4
	Min–max	0.03–18.45	0.03–9.81	0.01–1.67	0.009–0.49
im vs ad					
U		0	1.0	NS	0
P		0.001	0.001		0.01
M vs F					
U		NS	NS	NS	NS
P					

*im* immature, *ad* adult, *M* male, *F* female, *AM* arithmetic mean, *SD* standard deviation, *Med* median; *CV* coefficient of variation in percent, *M*–*W U* Mann–Whitney *U* test, *p* level of significance, *NS* difference nonsignificant

Between adult males (ad M) and females (ad F) there were no statistical differences in the Hg concentrations in the corresponding tissues. Accordingly, immature individuals were combined into one group (im F + im M), similarly to adults (ad F + ad M). Comparisons of Hg concentrations in biological materials showed that in the adults Hg concentrations in the liver (*p* < 0.001), kidney (*p* < 0.001), and brain (*p* < 0.01) were higher than in the immature individuals (Mann–Whitney *U* test; *p* < 0.05) (Table 1). In the liver, kidney, and brain, Hg concentrations in adult raccoons were 53, 19, and 11 times higher than in the immature raccoons, respectively. In the immature and adult raccoons, muscle Hg concentrations were not statistically significant.

Table 2 presents the results of comparisons between Hg concentrations in the examined tissues within and between adult and immature groups. The adult group showed differences in Hg concentrations between most tissues with the exception of the liver vs kidney and muscle vs brain. In the immature group, there was a significant difference in Hg concentration between kidney and brain (*p* < 0.05). Comparing the immature to adult groups, we found differences in Hg concentrations between the liver and brain (*p* < 0.01) and between the brain and kidney (*p* < 0.001) (Kruskal–Wallis test; *p* < 0.05).

**Table 2:** Evaluation of the significance of differences in mercury concentrations between the examined materials collected from raccoons, depending on the age group

K–W	L vs K	L vs M	L vs B	K vs M	K vs B	M vs B
Adultus						
*p*	NS	0.01	0.001	0.01	0.0001	NS
Immaturus						
*p*	NS	NS	NS	NS	NS	0.04
Adultus and Immaturus						
*p*	NS	NS	0.01	NS	0.001	NS

*L* liver, *K* kidney, *M* muscle, *B* brain, *KW* Kruskal–Wallis test, *p* level of significance, *NS* not significant

Analysis of *r*_*s*_, which used data from all individuals (*n* = 24), showed that the strongest relationships (*r*_*s*_ > 0.90) existed between the liver and kidney (*r*_*s*_ = 0.98, *p* < 0.001), liver and brain (*r*_*s*_ = 0.92, *p* < 0.001), and the brain and kidney (*r*_*s*_ = 0.91, *p* < 0.001). Weaker relationships existed between skeletal muscle and liver (*r*_*s*_ = 0.52, *p* < 0.01) and between skeletal muscle and kidney (*r*_*s*_ = 0.57, *p* < 0.05).

Additionally, the adult raccoons (*n* = 17) showed statistically significant (*p* < 0.001) and positive correlation coefficients between liver and kidney Hg concentrations (*r*_*s*_ = 0.98), between kidney and muscle Hg (*r*_*s*_ = 0.87), between liver and brain Hg (*r*_*s*_ = 0.96), and slightly weaker but also significant (*p* < 0.05) correlations between Hg concentrations in the kidney and brain, muscle and brain (in both cases, *r*_*s*_ = 0.81). In the immature raccoons (*n* = 6), there was only one significant relation (*p* < 0.05)—between Hg concentration in the liver and kidney (*r*_*s*_ = 0.81).

A strong relation between Hg concentrations in the tissues of raccoon was observed also for all the males, i.e., immature and adult (im + ad) and all females (im + ad). In males, the highest Spearman’s correlation coefficient was found between the Hg concentrations in the liver and kidney, liver and brain (in both cases: *r*_*s*_ = 0.98, *p* < 0.001), kidney and brain (*r*_*s*_ = 0.95, *p* < 0.001), and slightly lower between Hg concentrations in the liver and muscle, and kidney and muscle (*r*_*s*_ = 0.65 and *r*_*s*_ = 0.64, *p* < 0.01, respectively). In the group of females, we found a relation between the kidney and liver Hg levels (*r*_*s*_ = 0.98, *p* < 0.001), as well between the liver and brain (*r*_*s*_ = 0.90, *p* < 0.05).

## Discussion

Raccoons are deemed good bioindicators of environmental contamination, especially in the USA and Canada. As omnivores, they are susceptible to the biomagnification of Hg due to the consumption of contaminated lower-trophic level organisms from aquatic and terrestrial food chains [20, 22]. Ecotoxicological studies using raccoons, conducted mainly in North America, have most frequently examined the liver and kidney (organs with the main role in detoxification), and less frequently the muscle and brain (Table 3).

**Table 3:** Comparison of mean mercury concentrations in the liver, kidney, muscle, and brain of raccoon from Poland and North America (mg/kg dw and ww; in the dw and ww conversions, it was found that both the liver and muscle contained 70 % water and the kidney and brain contained 80 % water)

Liver (L)	Kidney (K)	Muscle (M)	Brain (B)	Dry/wet weight	Number (*n*)	Country	Source
2.99	2.07	0.50	0.14	dw	24	Poland, WMNP	this study
0.90	0.41	0.15	0.03	ww			
–	–	0.43	–	dw	3 M	USA, Georgia,	[40]
–	–	0.13	–	ww		Piedmont	
7.8	–	0.73	–	dw	6 L/6 M	USA, Upper Coastal	
2.34	–	0.22	–	ww			
4.77	–	0.93	–	dw	4 L/22 M	USA, Lower Coastal	
1.43	–	0.28	–	ww			
15.1	–	1.3	–	dw	4 L/5 M	USA, SCSRS	
4.53	–	0.39	–	ww			
4.5	5.47	1.1	–	dw	95	USA, SCSRS: Steel Creek delta	[20]
1.35	1.64	0.33	–	ww			
23.4	–	–	2.1	dw		USA, California	[57]
7.02			0.63	ww			
1.37	1.20			dw	15	USA, Macon Country, Alabama	[36]
0.41	0.24	–	–	ww			
4.43	–	–	–	dw	47	USA, SRS	[58]
1.33	–	–	–	ww			
4.83	3.93	–	–	dw	70	USA, SRS	[42]
1.45	1.18	–	–	ww			
28.2	–	–	–	dw	4	USA, SRS	[27]
8.46	–	–	–	ww		NWR, Hutchinson Island	
7.2	–	–	–	dw	3		
2.16	–	–	–	ww		Control site	
54.0	5.0	1.9	0.96	dw	11	USA, South Florida	[21]
16.2	1.49	0.57	0.29	ww		Everglades	
0.12–0.68	0.25–1.95	–	–	dw	16	USA, Central New York	[38]
0.04–0.21	0.05–0.39						
				ww			
6.7	6.8	–	–	dw		USA, Wisconsin	[37]
2.01	1.37			ww			
13.66	–	2.44	–	dw	5	USA, Texas and Louisiana	[59]
4.1		0.73		ww			
–	0.055	–	0.575	dw	10	USA, Emory River, East Tennnessee	[22]
–	0.115	–	0.011	ww			
15.1	3.7	0.93	–	dw	4	Canada, Georgia Bay	[9]
4.53	1.11	0.28		ww			
3.93	1.35	–	0.13	dw	25	Canada, SNWR	[35]
1.18	0.27		0.04	ww			

*WMNP* Warta Mouth National Park, *SNWR* Shiawassee National Wildlife Refuge, *SCSRS* South Carolina Savannah River Site

The availability of Hg appears to be more associated with river/wetland habitats where methylation processes are enhanced, as seen in the significant differences in Hg levels in raccoons from the river/wetland systems compared to more upland areas [27]. Without comprehensive species- and site-specific data on Hg contamination, it is difficult to assess the level of risk to animals inhabiting a particular ecosystem [28]. Based on a review of available literature, the US Fish and Wildlife Service proposed a level of 1.1 mg/kg ww (5.5 mg/kg dw) for liver, kidney, blood, and hair in wild mammals that “should be considered as presumptive evidence of an environmental mercury problem” [3].

Levels of exposure that induce Hg poisoning in mammals vary among species. Threshold Hg concentrations for the raccoon (accumulation resulting from the geochemical background) have not yet been established, likewise sublethal and lethal concentrations. Laboratory tests and observations conducted on the domestic dog (*Canis familiaris*) show that typical Hg levels (reflecting their geochemical background) in the liver and kidney of this domesticated mammal are <0.1 mg/kg ww (~0.3 mg/kg dw) and lethal Hg concentration to these organs are at 2.8 and 3.3 mg/kg ww (9.3 and 11 mg/kg dw), respectively [29]. In the examined raccoon from WMNP Poland, the concentrations of Hg in the kidney and liver were approximately 10 and 7 times higher than the values reflecting the domestic geochemical background suggested by Farrar et al. [29].

### Mercury in Liver and Kidney

The liver is the major site of MeHg biotransformation. In raccoons from the Everglades National Park, Hg concentrations in the liver were more than 18 times higher than in individuals from NW Poland [21] (Table 3). Elevated concentrations of this element in the late 1980s and 1990s in many animals from the Everglades were associated with air pollution from metal mining and smelting, coal-fired utilities and industry, and solid-waste incinerators [30, 31].

Mercury concentrations in the liver in the examined raccoons living in the Warta Mouth National Park in northwestern Poland ranged from 0.03 to 18.45 mg/kg dw (mean, 2.99 mg/kg dw), with the concentrations of four specimens considered elevated (>5.5 mg/kg dw). The results do not indicate a risk of death from acute Hg intoxication as the measured levels were not even close to the 125 mg/kg dw (25 mg/kg ww) reported in kidneys of carnivorous mammals, the level associated with lethal mercury poisoning [32].

The kidney may also play a role in the demethylation of MeHg, accounting for the high inorganic Hg concentrations in the kidney (55 %). High inorganic Hg in the liver and kidney relative to other tissues has also been reported for the river otter and the wild mink [33, 34]. Based on publications, kidney Hg concentrations in the North American raccoons range from 1.2 to 6.8 mg/kg dw. In individuals from the WMNP Hg concentration was generally low, with more than 54 % of the cases not exceeding 1 mg/kg dw, and concentration ranges of 1–4 and >5 mg/kg dw accounted for 30 and 16 % of the samples, respectively.

Biomonitoring studies using raccoons have been carried out in the Shiawassee National Wildlife Refuge (SNWR) in Canada [35]. In the SNWR, raccoons were acquired from the backwaters of rivers flowing through the Refuge, the quality of which is generally poor. Most of the rivers also pass through cities, heavily industrialized areas, and agricultural land, and therefore are exposed to industrial and wastewater discharge, and agricultural runoff. Mercury concentrations in the liver, muscle, and brain tissues in raccoons from the Canadian Refuge and the Warta River Mouth National Park, Poland were similar, and in both cases the Hg concentration in adults was greater in adults than in young individuals. Comparison of Hg concentrations in the liver, kidney, and brain tissues of raccoons from the SNWR in Canada [35] between the two age categories (immatures and adults) showed a relation similar to that observed in individuals from the WMNP in Poland. The mercury concentrations in the studied organs were higher in adults than in young individuals. Mercury levels in the kidney in adults from Canada were lower than levels reported in this research. The results from this study are consistent with those reports that indicate the liver and kidney as the major storage sites for Hg in raccoons, followed by the muscle and brain. Furthermore, they also confirm the age-related upward trend in the concentration of toxic metals in the liver and kidney of raccoons. In raccoons from NW Poland, similar to raccoons from Alabama in the USA [36] and the SNWR in Canada [35], Hg concentration in the liver and kidney did not show any significant differences between genders. High Hg concentrations in the kidney and liver of raccoons (6.8 and 6.7 mg/kg dw, respectively) were found by Sheff and Amant [37]. Although those studies were conducted in uncontaminated sites near Wisconsin, they were located in areas where Hg occurs naturally at high levels in soil and rock.

In most cases, liver and kidney Hg levels in raccoon from Poland were lower than those found in the USA and Canada (Table 3), at approximately 3 and 2 mg/kg dw. However, they were greater than in the population of Central NY and Macon Country, Alabama (USA) [36, 38].

Our previous studies carried out in the Warta Mouth National Park [11] showed that Hg concentrations in the liver and kidney in American mink (*N. vison*) were 11.8 and 14.1 mg/kg dw, almost four and seven times higher than in the raccoon in this study, respectively. These significantly higher liver and kidney Hg concentrations in mink compared to raccoon in the same area are probably related to differences in the diet of the animals. Raccoons in the WMNP eat mainly small mammals (primarily rodents), which account for 40 % of their diet, while fish and amphibians comprise only 13 %. The American mink is a very piscivorous species—in the WMNP its diet also includes piscivorous birds [17]. According to a study by Kucera [39], the American mink occupies a top trophic position in the aquatic foodweb and bioaccumulates Hg from food, about 10 times more Hg on a concentration basis than predatory fish from the same drainage areas.

### Mercury in Muscle

In studies conducted in different regions of the eastern USA, it was found that muscle Hg levels were the lowest in Piedmont raccoons (0.43 mg/kg dw), higher in raccoons from the Upper Coastal Plain (0.73 mg/kg dw) and the highest in Lower Coastal Plain raccoons (0.93 mg/kg dw) [40]. However, the highest Hg concentration those authors reported in raccoons from the US Atomic Energy Commission Savannah River Site (SRS) in South Carolina was in skeletal muscle and the liver, 1.3 and 15.1 mg/kg dw, respectively. Compared to the SRS, Hg concentrations in the liver and muscle of WMNP raccoons were five and two times lower, respectively, which may result from the significant industrial Hg pollutant in the said part of the SRS. The nearby Savannah River flows below the principal sources of Hg at Augusta, Georgia, and was used for atomic reactor cooling at the SRS. During the past 50 years, Hg and other contaminants have been released into the SRS environment from various sources [41]. That region had been used for nuclear weapons production, and in the past levels of several toxic elements, including Hg in water, were considerably excessive [42], as is shown in numerous reports on raccoons [20, 27, 40, 43]. In addition, the diet of North American and Canadian raccoons is rich in fish, frogs, and birds which may contain significant amounts of Hg [44].

In contrast to Europe, the North American raccoon is hunted not only for its fur but also for its meat. Therefore, a significant part of the raccoon carcass is a potential source of Hg (in muscle mainly as MeHg), toxic to humans, predatory and scavenging birds, and mammals [45]. For these reasons, it is reasonable to determine Hg in raccoon muscle, especially in ecotoxicological studies. Mean Hg concentrations in the muscle of raccoons in North American areas slightly contaminated with Hg are less than 1 mg/kg dw (0.4–0.9 mg/kg dw; Table 3). The highest Hg concentrations (~2 mg/kg dw) were recorded in the muscles of the raccoon from Caddo Lake (Texas and Louisiana, USA) and Everglades Park, which resulted from a significant Hg pollution (Table 3).

### Mercury in Brain

Organic and inorganic Hg compounds easily permeate the blood–brain barrier [46, 47]. Although Hg toxicity primarily affects the nervous system, environmental analyses seldom concern Hg in mammalian brains [5]. However, in some reports, the brains of rats experimentally exposed to incremental doses of Hg showed increasing pathomorphological changes, including vascular lesions, disappearance and demyelination of neurons, necroses in the cerebellum cortex and brain stem nuclei, and atrophy and abnormal density of granulosa cells of the cerebellum [48–50]. Moreover, Basu et al. [51] observed significant neurochemical changes in wild mink, although brain THg (total mercury) levels were low, 0.11 to 13.4 mg/g ww (0.22–26.8 mg/g dw), i.e., below the concentrations that may cause adverse effects as proposed by the US EPA [52].

Out of 13 papers concerning field studies on raccoons in North America, only 4 included information on Hg concentration in the brain. In individuals from the WMNP in Poland, brain Hg concentration was low (0.14 mg/kg dw), much less than the low limit given by Basu et al. [51]. In raccoons from different parts of North America, reported mean brain Hg concentrations are generally higher than in Poland and range from 0.13 to 2.1 mg/kg dw (Table 3). The highest Hg concentrations were found in the brain of raccoon from California, more than 15 times higher than in the WMNP. However, Souza et al. [22] found a more than two times lower Hg concentration in the brain of raccoon in East Tennessee, in an area exposed to coal fly ash (Table 3), where more than five million cubic yards of coal fly ash and water spilled into the Emory River in 2008, significantly polluting it with oxides of silicon, aluminum, iron, and trace elements [53]. Low Hg concentration in the brain of raccoons could have resulted from the fact that the coal fly ash had limited amounts of Hg and other hazardous metals.

### Toxicity

In animals, the effects of Hg toxicity are manifested by reduced reproductive success associated with embryo mortality and, in particular, damage to the central nervous system including the cerebellum, ataxia and paresthesia, described in literature as the “dancing cats” illness. Among raccoons, Hg poisoning is very rare. However, fatal Hg poisonings have been described in panthers (*Puma concolor coryi*) from the Florida Everglades, Shark River Slough [54]. The normal diet of panthers from the Everglade Park comprises deer and wild hog, but raccoons may also become a major component of the panther’s diet. Roelke [54] reported that raccoons from the same area had Hg levels from 10 to 100 times greater than deer. It was found that Hg concentration in the liver of dead panther ranged from 26 and 110 mg/kg ww. In comparison, Charbonneau et al. [55] reported that cats chronically fed with lethal concentrations of MeHg had mean total Hg concentrations in the liver ranging from 38 to 92 mg/kg ww. These elevated Hg levels in panthers have been correlated with elevated levels of total Hg in their prey; raccoons, and alligators [31, 54]. Mercury levels found in the muscle of raccoons from the greater Shark River Slough area were well above 1.0 mg/kg ww (range, 1.01–1.80 mg/kg ww) and were significantly higher (*p* < 0.05) than in raccoons from other areas examined [56]. The accumulation of Hg in raccoons largely depends on diet, especially the portion of fish, as documented in several cases. Raccoons in the same area as the panthers in Florida had nine times greater Hg concentrations in their muscle than individuals from the WMNP.

### Sensitivity

Comparing Hg concentrations in the organs and tissues of raccoons from northwestern Poland, the highest was found in the liver, lower in the kidney and muscle, and the lowest in the brain. These observations are consistent with data of other authors on the content and distribution of this element in the raccoon from North America and Canada (Table 3). For example, Porcella et al. [21] observed that raccoon in North America had Hg concentration in the liver greater than in the kidney. Similarly, Wren et al. [28] noted that the Hg concentration in the liver of raccoons from Georgia Bay in Canada was four times higher than in the kidneys.

Scant existing publications on Hg concentrations in the raccoon have described relations between concentrations of this metal determined in various animal tissues. Lord et al. [20] found a correlation between the concentrations of Hg in the liver and kidney, and the liver and muscle (*r* = 0.60). Porcella et al. [21] documented the existence of a stronger relationship between the liver and kidney (*r* = 0.71), suggesting that the liver is the source of Hg to the kidney or that the kidney demethylates MeHg as well. Analysis of our results showed the existence of strong relations between Hg concentrations in the liver and kidney, liver and brain, and brain and kidney (*r*_*s*_ ≈ 0.90), and weaker between Hg concentrations in the liver and muscle, and kidney and muscle (*r*_*s*_ ≈ 0.50).

Our study is analogous to some investigations in North America that have been conducted for many years. Our results on raccoons living near a large Polish river were similar to reports by Lord et al. [20] and Souza et al. [22] who also investigated raccoons living in riparian areas with relatively low Hg pollution. In other parts of North America, with higher Hg contamination, raccoons showed high Hg concentrations.
